# Nestin Positive Bone Marrow Derived Cells Responded to Injury Mobilize into Peripheral Circulation and Participate in Skin Defect Healing

**DOI:** 10.1371/journal.pone.0143368

**Published:** 2015-12-03

**Authors:** Yi Yang, Danlin Pang, Chenghu Hu, Yajie Lv, Tao He, Yulin An, Zhangui Tang, Zhihong Deng

**Affiliations:** 1 State Key Laboratory of Military Stomatology, Center for Tissue Engineering, School of Stomatology, Fourth Military Medical University, Xi’an, Shaanxi, China; 2 Xiangya Stomatology Hospital, Central South University, Changsha, Hunan, China; 3 Department of Otolaryngology, Xijing Hospital, Fourth Military Medical University, Xi’an, Shaanxi, China; 4 Xi’an Institute of Tissue Engineering & Regenerative Medicine, Shaanxi, China; 5 Department of Dermatology, Tangdu Hospital, Fourth Military Medical University, Xi’an, Shannxi, China; 6 Department of Oral Prosthodontics, School of Stomatology, Fourth Military Medical University, Xi’an, Shaanxi, China; Children's Hospital Boston/Harvard Medical School, UNITED STATES

## Abstract

Exogenously infused mesenchymal stem cells (MSCs) are thought to migrate to injury site through peripheral blood stream and participate in tissue repair. However, whether and how endogenous bone marrow MSCs mobilized to circulating and targeted to tissue injury has raised some controversy, and related studies were restricted by the difficulty of MSCs identifying *in vivo*. Nestin, a kind of intermediate filament protein initially identified in neuroepithelial stem cells, was recently reported as a credible criteria for MSCs in bone marrow. In this study, we used a green fluorescent protein (GFP) labeled bone marrow replacement model to trace the nestin positive bone marrow derived cells (BMDCs) of skin defected-mice. We found that after skin injured, numbers of nestin^+^ cells in peripheral blood and bone marrow both increased. A remarkable concentration of nestin^+^ BMDCs around skin wound was detected, while few of these cells could be observed in uninjured skin or other organs. This recruitment effect could not be promoted by granulocyte colony-stimulating factor (G-CSF), suggests a different mobilization mechanism from ones G-CSF takes effect on hematopoietic cells. Our results proposed nestin^+^ BMDCs as mobilized candidates in skin injury repair, which provide a new insight of endogenous MSCs therapy.

## Introduction

Mesenchymal stem cells (MSCs) are greatly potential in tissue injury repair due to their multilineage differentiation ability, immunomodulatory capacity and trophic function [1–2]. Although local transplantation and systemic infusion of MSCs have been treated as effective cellular therapy in multiple models of injury from heart, brain to bone and cartilage, it attracted attribution in particular that if endogenous MSCs could be mobilized and targeted to injury site to participate in tissue repair [3–4]. This method does not require in vitro culture process and may avoid the high loss ratio of infused MSCs in circulation [5–7], but whether and how endogenous MSCs can be mobilized has raised some controversy.

Growing evidence has shown that systemic infused MSCs migrate to host organs including heart, liver, spleen and bone marrow, where they mostly reside in initially [8–12]. Moreover, the infused MSCs perform a significant tendency to home to injury tissues compared with other organs, which is thought to be the first step for MSCs to participate in tissue repair [13]. Whereas, there was a consistently debate whether endogenous bone marrow MSCs can be mobilized to peripheral blood and injury site. Several early studies successfully isolated adherent fibroblast-like cells from normal peripheral blood which had differentiation potential of osteoblasts and adipocytes, and did not express haemopoietic markers such as CD34 or CD45 [14–16]. Other studies, on the contrary, failed to find these cells in peripheral blood at all [17–19]. Perhaps a wider accepted view is that there are few of these “blood derived multipotent mesenchymal stromal cells” in normal condition, but they are detectable in unsteady conditions including cancer, hypoxia or injury [20–24]. However, most of above mentioned studies did not clearly indicate the definite identity and origin of these cells. Some studies tried to prove these cells to be bone marrow MSCs by intra-bone marrow injection or re-implant of labeled MSCs and detecting them in blood or injured organs thereafter [25–26], but these are not direct evidences since whether the re-implanted cells could totally represent the host MSCs reside in bone marrow stem cell niches was doubtful. In our previous study, a chimeric mouse model whose bone marrow was destroyed by a lethal dose of irradiation and replaced with ones derived from GFP transgenic donor mice was constructed, and by tracing the distribution of GFP positive cells we illustrated that bone marrow derived cells (BMDCs) migrated to injure dental tissues mediated by stromal cell-derived factor-1 (SDF-1, also known as CXCL12) signal [27]. This study directly proved mobilization of endogenous BMDCs to injured tissue, but the definite component of these BMDCs was still unknown.

One of the challenges in studying of mobilized MSCs is the lack of a universally accepted marker for defining the MSCs phenotype, which makes identifying MSCs in vivo quite difficult and might be the reason for the conflicting results of previous studies [3, 5, 28]. Recent studies indicated nestin, a characteristic marker of multi-lineage progenitor cells first identified in neural stem cells, as a criteria of MSCs in bone marrow [29]. The nestin positive BMDCs represent a group of multipotent cells for multilineage differentiation and were negative for hematopoiesis markers. These nestin^+^ BMDCs are confirmed to be MSCs, and this finding facilitates tracing MSCs in vivo by providing a reliable phenotypic marker.

The nestin^+^ MSCs are components of hematopoietic stem cells (HSCs) niches that are important for HSC maintaining [29, 30]. They are spatially associated with HSCs and highly express HSCs maintenance genes including CXCL12, which could be significantly and selectively downregulated by G-CSF [29]. It is known that G-CSF mobilize HSCs through CXCL12-CXCR4 axis [31, 32]. Interestingly, quite a few studies observed inducement of circulating multipotent mesenchymal stromal cells [33–36] by G-CSF treatment. These findings triggered a hypothesis that the molecular mechanism of MSCs mobilization might resemble ones work in HSCs [21], but to date, there are few solid evidences for G-CSF’s effect on MSCs mobilization.

In this study, we used the GFP-positive bone marrow chimeric mice as models to trace the migration of nestin^+^ BMDCs after skin defect, analyzed their distribution in peripheral blood and injured skin, and observed G-CSF’s effect on mobilization of these cells.

## Materials and Methods

### Mice and wounding model

8-weeks old wild type and GFP^+/+^ transgenic female C57BL/6 mice were obtained from the Laboratory Animal Research Centre of the Fourth Military Medical University (FMMU). This study was approved by Fourth Military Medical University Intramural Animal Use and Care Committee and all interventions were performed in according with the committee guidelines for the use and care of experimental animals. All efforts were made to minimize suffering. Anesthetics were used as indicated.

Mice were anesthetized by intraperitoneal administering 1% pentobarbital. Once adequate anesthesia was obtained, a 6 mm or 20 mm wound was punched in the depilated dorsal skin. Wounds were left uncovered, and mice were housed individually with sterile paper bedding. Mice were given analgesic and antibiotics and allowed to recover. Animals (4–7/group) were sacrificed 4 and 7 days after wounding. Biopsies containing the wounded tissue and the surrounding skin were harvested. At the conclusion of the study period or prior to the harvesting of mouse wounds, peripheral blood or bone marrow, all animals were euthanized by CO_2_ narcosis.

### Bone marrow transplantation

Wild type C57BL/6 mice were used as recipients and received a disposable whole body Co^60^ ionizing radiation of 8.5 Gy in dosage. The donor GFP transgenic C57BL/6 mice were sacrificed and the femurs and tibias were isolated. Then the epiphyseal sides of the bones were cut off and the whole bone marrow tissue was flushed out with PBS by a 1ml injector. Red blood cell lysis buffer (CWBio, Beijing, China) was used to remove the erythrocytes. Then 2×10^6^ of bone marrow cells were suspended in 100 μl PBS and injected through tail vein to each recipient mouse within 6 hours after the irradiation. Then 2 x 10^6^ GFP^+^ bone marrow cells were suspended into 100 ml PBS and injected intravenously by tail vein into wild-type C57BL/6 mice within 24 h after 8.5 Gray of whole body irradiation. The successful creation of GFP chimera was confirmed by analysis of the GFP^+^ peripheral blood mononuclear cells (PBMCs) by flow cytometry (FACS Calibur; BD, San Jose, CA). The percentage of GFP^+^ PBMCs in recipients quickly increased and reached 85% by 30 days (data was not shown), and then the chimera mice were used in the study.

### G-CSF administration

24 chimeric mice received an intravenous injection of G-CSF (Qilu-Pharma, Shandong, China) through tail veins for continuous 5 days after the skin surgery (10ug/kg weight/dose), while 12 chimeric mice received same volume of normal saline as control.

### Histological and immunofluorescent analysis

To assess the healing process of skin defect, samples of injured skin were collected on day 7 after surgery for H&E staining was performed as described before [37]. Samples of injured skin, healthy skin, heart, liver, thymus and pancreas were collected on day 4 after surgery for immunofluorescent analysis of GFP^+^/nestin^+^ cells distribution.

### In vivo fluorescence imaging

A Xenogen In Vivo Imaging System (IVIS; Caliper Life Sciences, Hopkinton, MA, USA) was used to image and analyze the distribution of GFP^+^ cells of the chimeric mice 1 hour or 4 days after surgery following the instruction manual. A region of interest (ROI) of 1cm in diameter for each mouse was drawn to contain the whole area of radiation signal around the skin injury, and the results were expressed as total fluorescence signal efficiency (total units of photons per second within the ROI).

### RT-PCR

On day 7 after the skin defect was made, the full thickness of injured skin tissue together with 3mm width of healthy skin around was isolated from each sample, and total RNA was isolated using Trizol reagent (Invitrogen, Carlsbad, CA, USA). Reverse transcription of mRNA was possessed with a PrimeScript RT reagent kit (TaKaRa, Dalian, China) and RT-PCR was performed using the QuantiTect SYBR Green PCR Kit (Toyobo, Osaka, Japan) and detected on the ABI Prism 7500 HT sequence detection system (Applied Biosystems, Foster City, CA, USA). GAPDH was used as internal control gene and the primer sequences were as follow: CXCL12, Forward, 5’-GAGCCAACGTCAAGCATCTG-3’, Reverse, 5’- CGGGTCAATGCACACTTGTC-3’; GAPDH, Forward, 5’-AGCAGTCCCGTACACTGGCAAAC-3’, Reverse, 5’- TCTGTGGTGATGTAAATGTCCTCT -3’.

### Statistical analysis

Data are presented as means ± SD from at least three independent experiments. Statistical comparison of two groups was carried out by two-tailed unpaired Student's t test using SPSS 17.0 software.

## Results

### Quantities of nestin positive cells in peripheral blood and bone marrow both increase after skin defect

**Fig 1:**
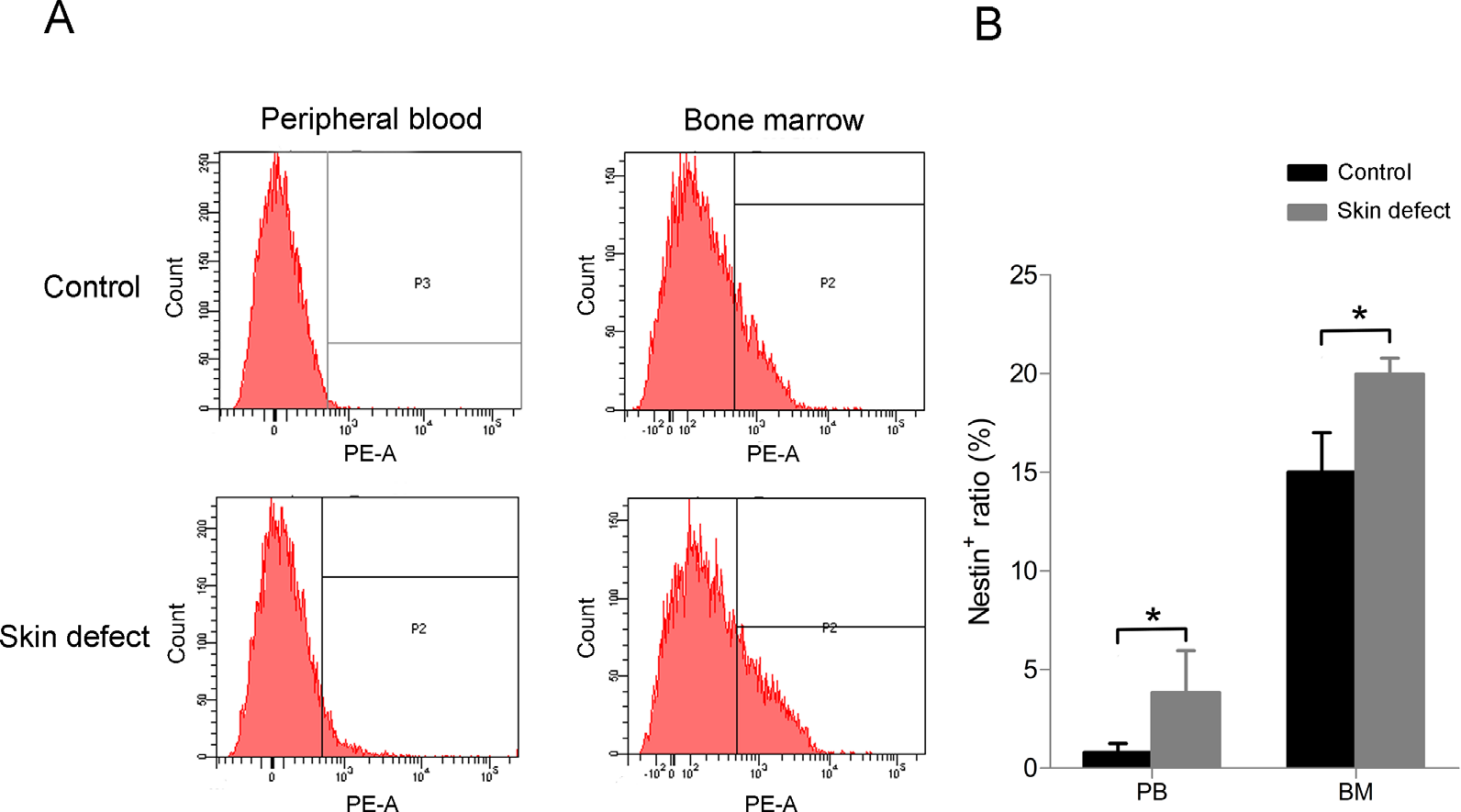
Quantity of nestin positive cells in peripheral blood (PB) and bone marrow (BM) both improve when there is an acute skin defect. (A) The result of flow cytometry and (B) the statistical analysis. Data (± SD) are representative of three independent experiments. Student’s t test was performed to determine statistical significance (*p<0.05, n = 7).

To investigate the change in quantity of nestin positive cells upon acute injury, a circular full thickness skin defect of 6 mm in diameter was made on the back of C57BL/6 mice, and 4 days later the bone marrow tissue and peripheral blood samples were gathered, lysised of red cells and detected nestin positive ratio by flow cytometry. The results showed that quantity of nestin positive cells in peripheral blood was at a basal level lower than 1%, while skin injury raised this ratio for 4 times or more. Number of nestin positive cells in bone marrow tissue also increased after the operation (Fig 1).

### Nestin^+^ BMDCs are mobilized and recruited to injure site

**Fig 2:**
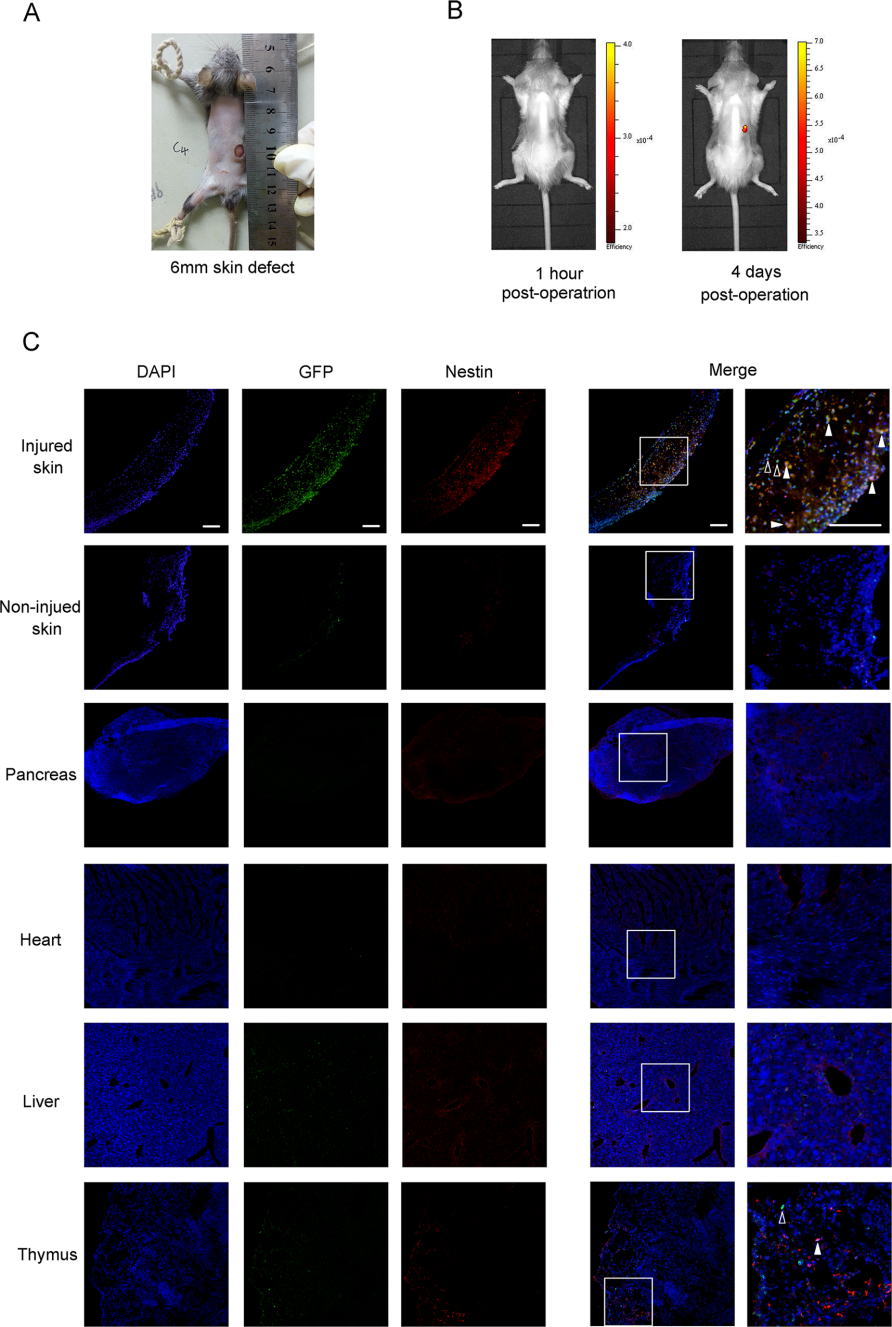
Nestin^+^ BMDCs are mobilized to the skin injury position. (A) General view of the just made 6mm full thickness skin defect. (B) In vivo fluorescence imaging of GFP^+^ BMDCs distribution 1 hour or 4 days after the skin defect made. (C) Immunofluorescence GFP^+^/nestin^+^ double positive cells around injured skin and other organs 4 days after operation. Solid arrows indicate the GFP^+^/nestin^+^ double positive cells and hollow arrows indicate GFP single positive cells. All scale bars, 100μm.

To test whether nestin^+^ BMDCs were mobilized when injury happens, a chimeric mouse model with GFP positive bone marrow derived cells was used. Wild type C57BL/6 mice received a lethal dose of ionizing radiation, in which case their own bone marrow tissues were destroyed and could be replaced with ones derived from GFP transgenic mice. After the bone marrow replacement, bone marrow derived cells of these chimeric mice were GFP positive while all of other cells were GFP negative (S1 Fig). 1 mouth later, skin defect was made (Fig 2A), and the trace of bone marrow derived cells could be observed by in vivo fluorescence imaging. The result showed that there was no significant accumulation of GFP^+^ cells one hour after the skin defect made, while 4 days later a notable concentration of GFP signal was observed at the position of injury (Fig 2B). Then we observed whether nestin^+^ BMDCs emerged at the injury position by immunofluorescence staining. The result manifested that nestin^+^ BMDCs were recruited to the skin wound and undetectable in uninjured skin tissue (Fig 2C). We also observed if thses cells spread in other organs including pancreas, heart, liver and thymus, finding that there are only several nestin^+^ BMDCs spread in thymus, while none of these cells could be observed in other organs at all. To investigate if these migrated cells promote injury recovery through pro-angiogenic function, we tested CD31 in injured skin tissue by immunofluorescence staining (S2 Fig). We found that expression level of CD31 in injured skin was higher than that in normal skin tissue, along with much more GFP^+^ BMDCs distributed around, and a small amount of GFP^+^/CD31^+^ double positive cells can be observed around injured skin tissue. These results suggested that nestin^+^ BMDCs were mobilized and specifically recruited to injure site when acute skin defect emerged, and might promote recovery via pro-angiogenic function.

### G-CSF fails to promote the mobilization of BMDCs toward injured skin, and takes no effect on injury repairing

**Fig 3:**
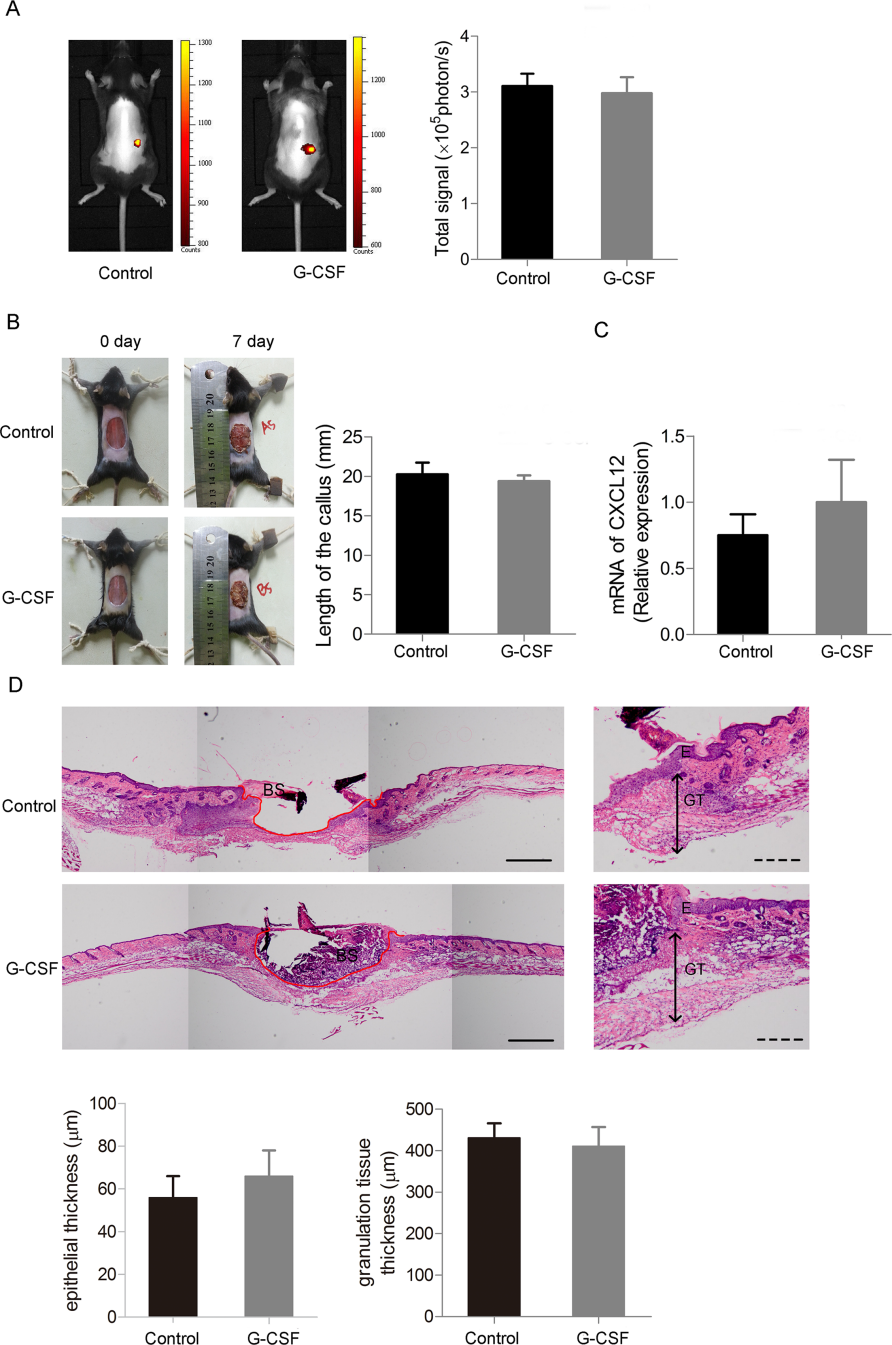
G-CSF does not promote mobilizing BMDCs and takes no effect on skin defect recovery. (A) In vivo fluorescence imaging analysis of GFP^+^ BMDCs distribution 4 days after the skin defect made given G-CSF or normal saline. (B) Left: General view of the 20mm skin defect 7 days after the operation given G-CSF or normal saline. Right: Statistics of callus length of the two groups. (C) RT-PCR of CXCL12 of the skin tissue from the two groups of mice. (D) Re-epithelialization of the injured skin of the two groups of mice. Upper: Representative HE staining images showing marginal location of the injured skin tissues. (The red lines indicate the outlines of the skin defects. E: epidermis, GT: granulation tissue and BS: blood scab.) Lower: Statistics of epithelial and granulation tissue thickness. Continuous scale bars, 500μm. Dotted scale bars, 200μm. (n = 4 per group). Data (± SD) are representative of three independent experiments. Student’s t test was performed to determine statistical significance (* p<0.05).

To investigate whether G-CSF could stimulate mobilization of bone marrow derived cells, we traced GFP positive cells of skin-injured chimeric mice given G-CSF or normal saline by in vivo imaging, finding no difference in fluorescence signal between the two groups (Fig 3A). To ensure whether G-CSF promote the healing progress of skin injury, a full thickness skin defect of ultimate size (20mm in diameter) was made before G-CSF administration. General view and HE staining showed that G-CSF failed to accelerate the re-epithelialization procedure of the wounds, and calluses of each group were of similar size during healing (Fig 3B and 3D). We also gathered skin tissue of the mice and detected mRNA expression level of CXCL12, an important chemotactic factor which is known to be highly relative with mobilization of BMDCs, also found no difference between the G-CSF treated mice and the control ones (Fig 3C).

### G-CSF improves quantity of nestin positive cells in bone marrow but not in peripheral blood

**Fig 4:**
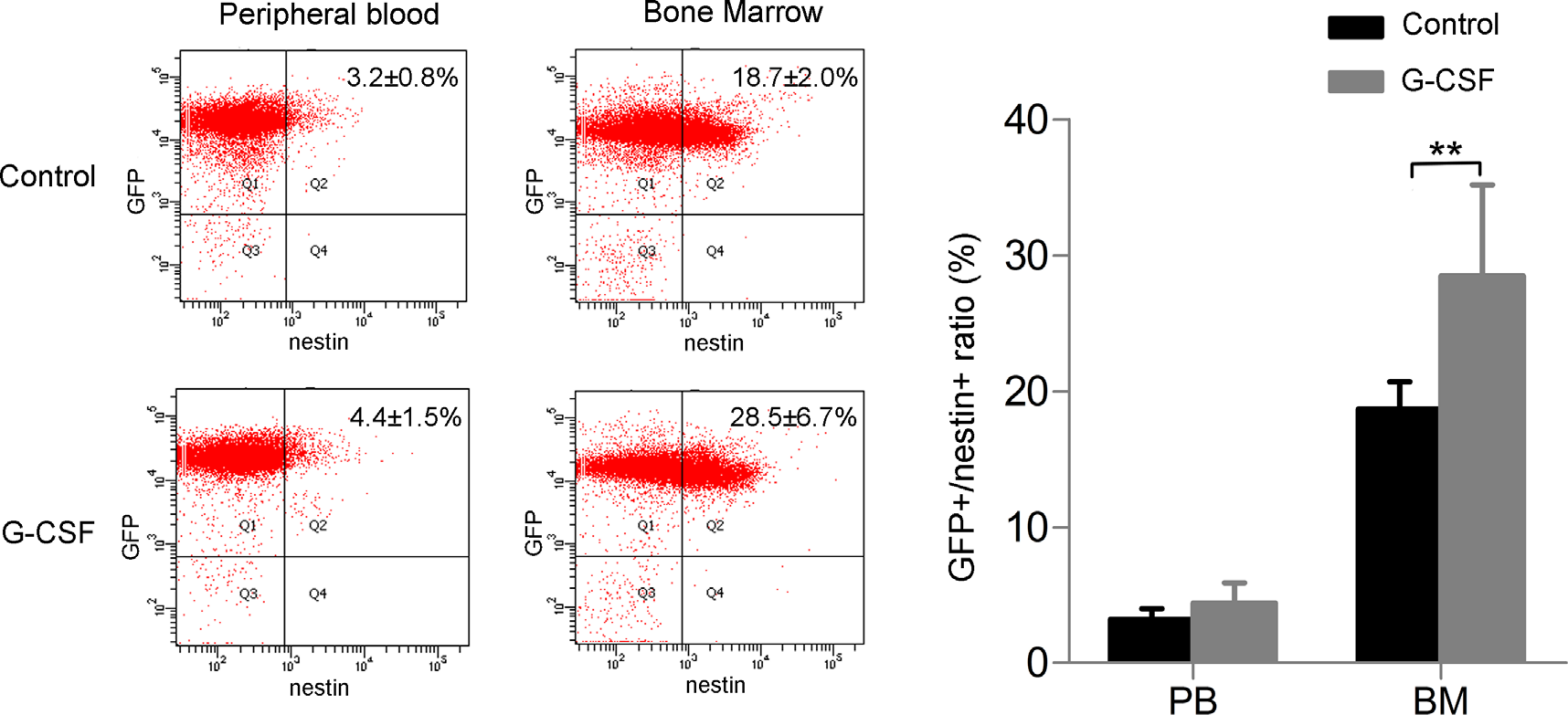
Quantity of nestin positive cells in bone marrow is increased by G-CSF after skin injury, but nestin positive BMDCs in peripheral blood does not change. (A) Flow cytometry of GFP^+^/nestin^+^ cells (showed in Q2) in peripheral blood and bone marrow given G-CSF or vehicle and (B) the statistical analysis. (n = 6 per group). Data (± SD) are representative of three independent experiments. Student’s t test was performed to determine statistical significance (**p<0.01).

Since no ameliorate effect of G-CSF on skin injury healing was observed, we then detected whether nestin positive cells in bone marrow could be mobilized by G-CSF to peripheral blood after skin defect. We check GFP and nestin positive cells in bone marrow and peripheral blood by flow cytometry after red cells lysising. Few GFP^-^/nestin^+^ cells were detectable either in bone marrow or in peripheral blood. Quantity of GFP^+^/nestin^+^ cells in bone marrow markedly increased when given G-CSF, but did not significantly change in peripheral blood (Fig 4). This phenomenon suggests that G-CSF does not promote the recruitment of nestin^+^ BMDCs to the injury.

## Discussion

MSCs are easily available and lack of immunogenicity, which permit local transplanting and systemic infusion of MSCs promising therapies for tissue regeneration. However, delivering and in vitro culture process of MSCs may have a significant impact on MSCs function and cause security problems [3]. Thus, it will be a non-invasive strategy avoiding these problems that if the host MSCs could be mobilized to peripheral blood and targeted to injury tissue. But since the lack of universally accepted criteria, studying of mobilized MSCs was complex and resulted in opposite outcomes. [3,5,28]. Nestin is a new identified characteristic marker of mesenchymal stem cells in bone marrow [29,38,39], and in this study, we use bone marrow chimeric mice for tracing nestin^+^ BMDCs and exploring MSCs mobilization after skin defect and G-CSF administration.

Circulating MSCs in peripheral blood are rare in quantity but may be concentrated by injury [23,24,40] or G-CSF [33–36] according to previous studies. In this study, we observed a significant increasing of nestin positive cells number in peripheral blood and bone marrow after skin defect (Fig 1). Moreover, there was a marked concentration of nestin^+^ BMDCs in injury site (Fig 2), while few bone marrow derived GFP^+^ cells were observed in non-defect skin and other organs (Fig 2C). These findings provide direct evidence that nestin^+^ BMDCs are mobilized to circulation and toward tissue injury. It was reported that MSCs can release angiogenic factors and protease to facilitate blood vessel formation, or might even differentiated into epithelial cells directly [41]. Our result confirmed the pro-angiogenic effect of BMDCs and might provide supporting evidence of this view since CD31^+^ BMDCs were found (S2 Fig).

It was reported that targeting the CXCR4-CXCL12 axis could increase endogenous bone marrow progenitor cells releasing into peripheral and stimulating diabetic wound healing [42]. However, G-CSF targeting CXCR4-CXCL12 axis did not promote this mobilization process in our system. Healing of the skin defect was not accelerated with G-CSF treatment (Fig 3), and number of circulating nestin^+^ BMDCs did not change (Fig 4). Notably, there were few nestin^+^/GFP^-^ cells in peripheral blood, suggested that the circulating nestin^+^ cells are mainly derived from bone marrow (Fig 4). In the beginning we used 6mm skin defect as ones made in Fig 2 to assess effect of G-CSF on wound healing, finding no difference in healing velocity or quality compared with the vehicle group (Data not shown). Since the healing speed of skin injury in mice is quite faster than other mammalian species, we inferred that G-CSF’s ameliorate function might be concealed in normal size of injury. Therefore, we made a larger skin defect of ultimate size which was 2cm in diameter, but still found no change in healing between G-CSF treated group and the control. CXCL12-CXCR4 axis plays import role in stem cells migrating including MSCs [43,44] and HSCs [45]. Fiorina et al. found that targeting the CXCR4-CXCL12 axis through anti-CXCR4 therapy could induce promoted HSC mobilization, which could promotes long-term survival of islet allografts. [46]. We checked mRNA expression of CXCL12 in injury skin tissue from G-CSF or vehicle treated mice and found no significant difference, which suggests that G-CSF does not mobilize bone marrow MSCs as it takes effect on HSCs. Interestingly, although G-CSF did not increase number of circulating nestin^+^ BMDCs, quantity of nestin^+^ cells in bone marrow raised markedly (Fig 4). Recent study reported that deletion of CXCL12 led to increased number of HSCs in blood and spleen without change of HSC number in bone marrow, which hints that CXCL12 is important for HSCs proliferation as well as retention in bone marrow, and deletion of CXCL12 leads to increased self-renewing division of HSCs [47]. We infer that G-CSF may promote MSCs proliferation by down-regulating CXCL12 in similar way, while for some reasons the proliferative MSCs would not depart from bone marrow or enter peripheral blood. Further research would be required to explain this phenomenon.

In summary, this study confirms the mobilization of nestin^+^ bone marrow MSCs into peripheral blood and toward injury skin, but meanwhile demonstrates that G-CSF does not promote MSCs mobilization and takes no effect on skin wound healing. These results suggest an undiscovered mobilization mechanism of MSCs that is different from ones work in HSCs, and further studies are expectable on this issue which is important for endogenous MSCs therapy in tissue repair.

## Supporting Information

S1 Fig The construction procedure of the GFP positive BMDC chimeric mice model. (A) Recipient mice received a disposable Co^60^ ionizing radiation of 8.5Gy, and the donor GFP transgenic mice were sacrificed and collected the whole bone marrow cells (WBMC), then the WBMC were lysised of red cells and immediately intravenous injected to the recipient mice within 6 hours after the radiation. One month later, the veinal blood of the recipient mice was gathered, lysised of red cells and detected GFP positive ratio by cytometry. The chimeric mice model was considered successfully constructed if the GFP positive ratio was over 85%. (B) The GFP positive ratio of the chimeric mice and the negative control.
(TIF)

S2 Fig Immunofluorescence of GFP positive cells and CD31 positive cells. Arrows indicate the GFP^+^/CD31^+^ double positive cells. All scale bars, 100μm.
(TIF)
