# Supplementary figures and images for: Nestin Positive Bone Marrow Derived Cells Responded to Injury Mobilize into Peripheral Circulation and Participate in Skin Defect Healing
Source: PLoS One. 2015 Dec 3;10(12):e0143368. doi: 10.1371/journal.pone.0143368 (PMC4669078; doi:10.1371/journal.pone.0143368)

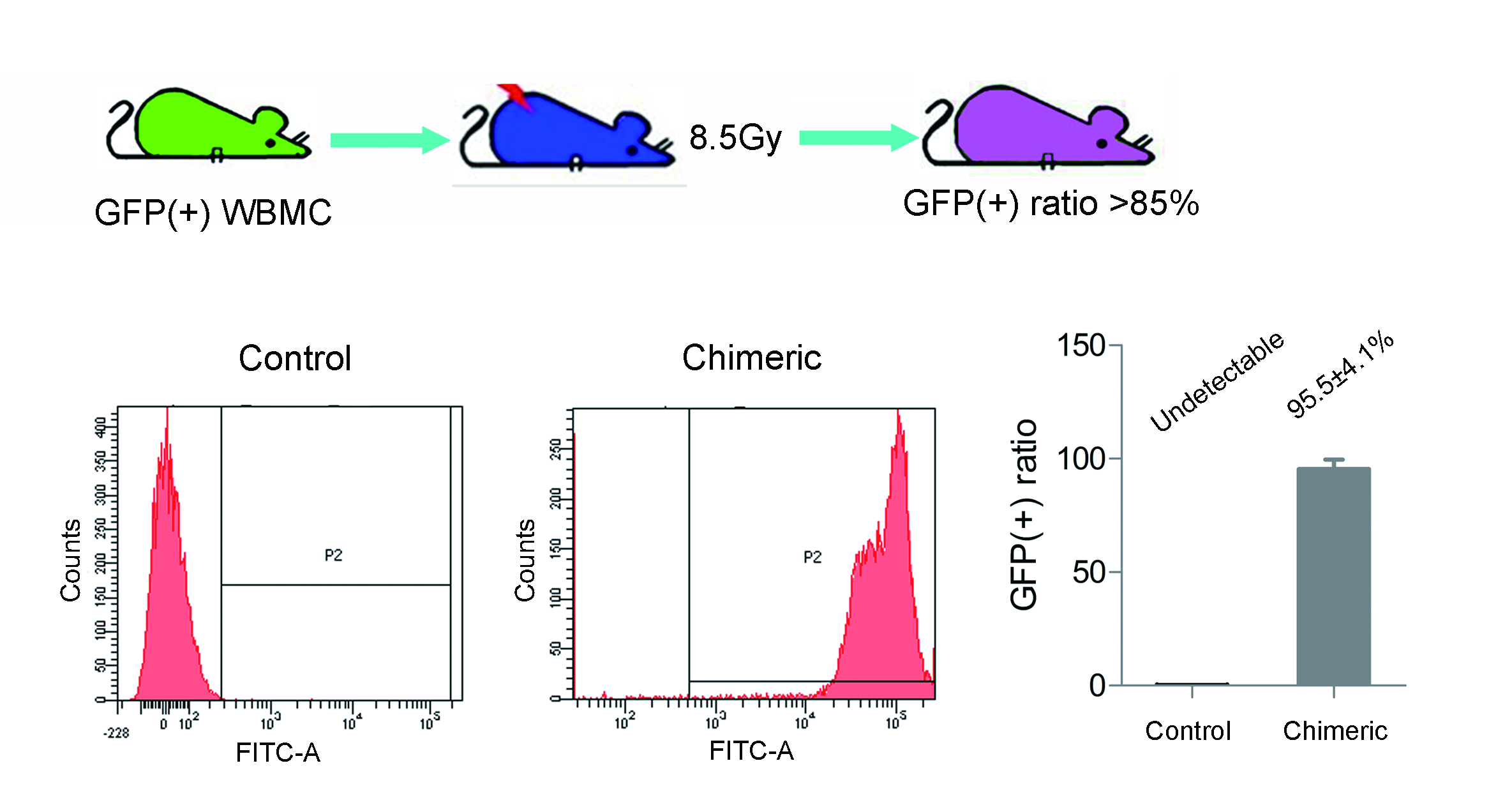

Supplement: S1 Fig — (A) Recipient mice received a disposable Co60 ionizing radiation of 8.5Gy, and the donor GFP transgenic mice were sacrificed and collected the whole bone marrow cells (WBMC), then the WBMC were lysised of red cells and immediately intravenous injected to the recipient mice within 6 hours after the radiation. One month later, the veinal blood of the recipient mice was gathered, lysised of red cells and detected GFP positive ratio by cytometry. The chimeric mice model was considered successfully constructed if the GFP positive ratio was over 85%. (B) The GFP positive ratio of the chimeric mice and the negative control. (TIF) [file pone.0143368.s001.tif]

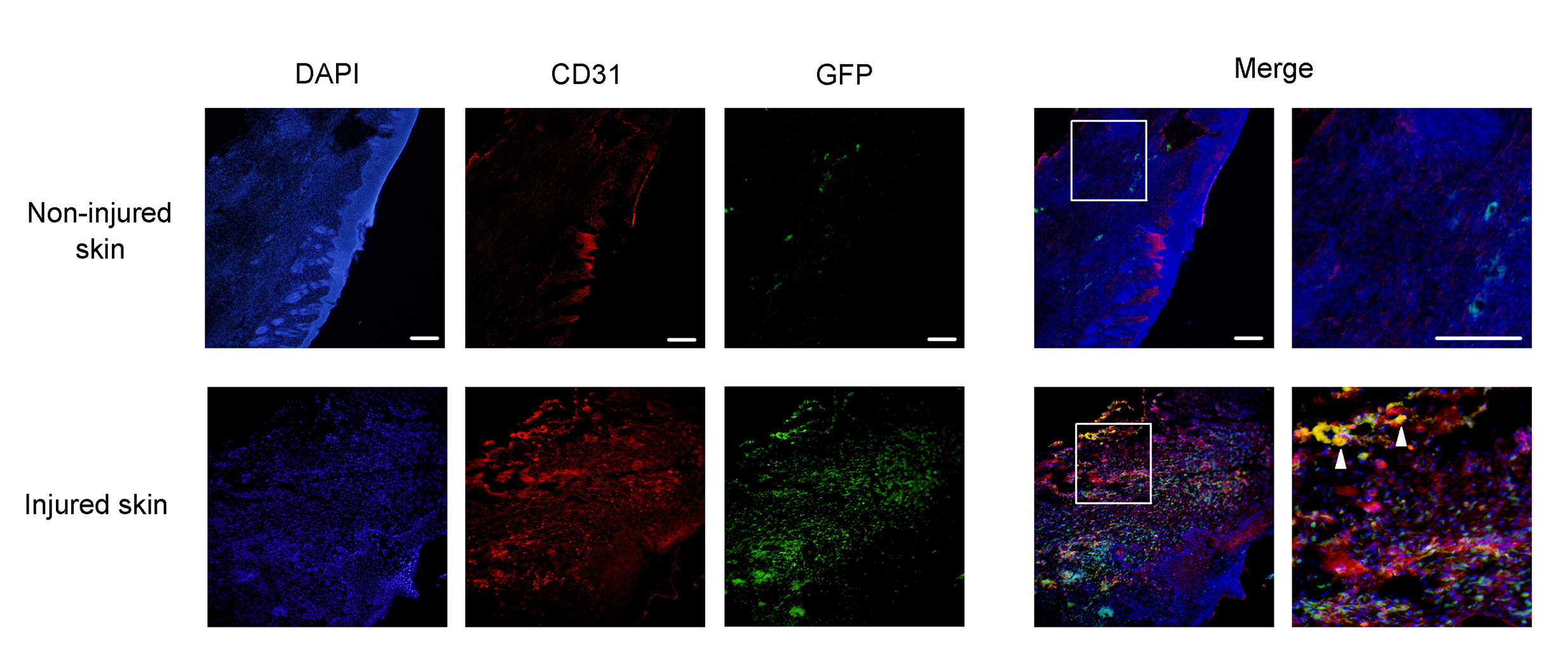

Supplement: S2 Fig — Arrows indicate the GFP+/CD31+ double positive cells. All scale bars, 100μm. (TIF) [file pone.0143368.s002.tif]
